# Supplementary material for: Genome-wide association study of cocaine self-administration behavior in Heterogeneous Stock rats
Source: Nat Commun. 2026 Jun 11;17:4876. doi: 10.1038/s41467-026-73694-w (PMC13261055; doi:10.1038/s41467-026-73694-w)
Supplement: Supplementary file 1 — Supplementary Information [file 41467_2026_73694_MOESM1_ESM.pdf]

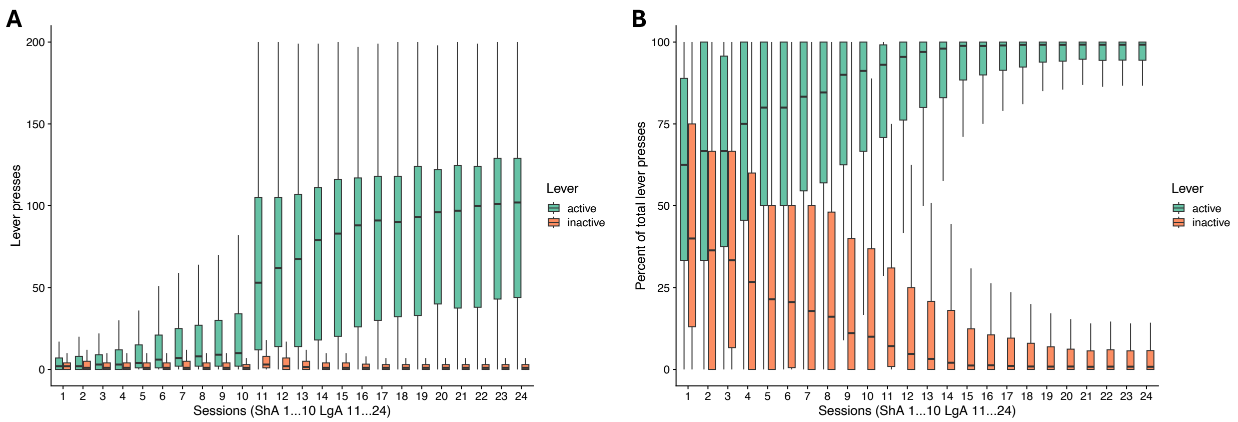

Supplemental Figure 1. Additional representation of active and inactive lever pressing. A) Box plots of the number of active and inactive lever presses for all animals across short and long access days (ShA 1-10 and LgA 1-14). B) Percent of active or inactive lever presses of the total presses during short and long access.
