# Supplementary material for: Genome-wide association study of cocaine self-administration behavior in Heterogeneous Stock rats
Source: Nat Commun. 2026 Jun 11;17:4876. doi: 10.1038/s41467-026-73694-w (PMC13261055; doi:10.1038/s41467-026-73694-w)
Supplement: Supplementary file 2 — Description of Additional Supplementary Files [file 41467_2026_73694_MOESM2_ESM.pdf]

## Description of Additional Supplementary Files

**Supplementary Data 1**: Description of all traits used for GWAS

**Supplementary Data 2**: Phenotype correlations among traits used for GWAS

**Supplementary Data 3**: Heritabilities with p values, n, and standard error for all traits used for GWAS

**Supplementary Data 4**: All genome-wide significant loci associated with traits used for GWAS, includes genomic position, minor allele frequencies, founder haplotypes

**Supplementary Data 5**: Corresponding genes in range for the genome-wide significant loci

**Supplementary Data 6**: Corresponding eQTLs for genome-wide significant loci

**Supplementary Data 7**: Corresponding sQTLs for genome-wide significant loci

**Supplementary Data 8**: Number of values imputed per rat across all sessions for short and long access (total <1% imputation across all sessions)

**Supplementary Data 9**: All raw and processed trait values used for GWAS

**Supplementary Code**: Full GWAS results including all genome-wide significant findings, Manhattan plots, and regional association plots
