# Supplementary material for: Genome-wide association study of cocaine self-administration behavior in Heterogeneous Stock rats
Source: Nat Commun. 2026 Jun 11;17:4876. doi: 10.1038/s41467-026-73694-w (PMC13261055; doi:10.1038/s41467-026-73694-w)
Supplement: Supplementary file 12 — Supplementary Code [file 41467_2026_73694_MOESM12_ESM.html]

montana\_cocaine\_redo\_GWAS


GWAS REPORT
