# Supplementary material for: Genome-wide association study of cocaine self-administration behavior in Heterogeneous Stock rats
Source: Nat Commun. 2026 Jun 11;17:4876. doi: 10.1038/s41467-026-73694-w (PMC13261055; doi:10.1038/s41467-026-73694-w)
Supplement: Supplementary file 13 — Reporting Summary [file 41467_2026_73694_MOESM13_ESM.pdf]

nature portfolio

Corresponding author(s): Olivier George, Abraham Palmer

Last updated by author(s): Apr 27, 2026

Reporting Summary

Nature Portfolio wishes to improve the reproducibility of the work that we publish. This form provides structure for consistency and transparency in reporting. For further information on Nature Portfolio policies, see our Editorial Policies and the Editorial Policy Checklist.

Statistics

For all statistical analyses, confirm that the following items are present in the figure legend, table legend, main text, or Methods section.

n/a	Confirmed	
☐	☒	The exact sample size (*n*) for each experimental group/condition, given as a discrete number and unit of measurement
☐	☒	A statement on whether measurements were taken from distinct samples or whether the same sample was measured repeatedly
☐	☒	The statistical test(s) used AND whether they are one- or two-sided<br>*Only common tests should be described solely by name; describe more complex techniques in the Methods section.*
☐	☒	A description of all covariates tested
☐	☒	A description of any assumptions or corrections, such as tests of normality and adjustment for multiple comparisons
☐	☒	A full description of the statistical parameters including central tendency (e.g. means) or other basic estimates (e.g. regression coefficient) AND variation (e.g. standard deviation) or associated estimates of uncertainty (e.g. confidence intervals)
☐	☒	For null hypothesis testing, the test statistic (e.g. *F*, *t*, *r*) with confidence intervals, effect sizes, degrees of freedom and *P* value noted<br>*Give P values as exact values whenever suitable.*
☒	☐	For Bayesian analysis, information on the choice of priors and Markov chain Monte Carlo settings
☒	☐	For hierarchical and complex designs, identification of the appropriate level for tests and full reporting of outcomes
☐	☒	Estimates of effect sizes (e.g. Cohen's *d*, Pearson's *r*), indicating how they were calculated

Our web collection on statistics for biologists contains articles on many of the points above.

Software and code

Policy information about availability of computer code

Data collection	Experimental behavioral assays and data collection are described in the methods section and additional detailed experimental protocols can be found at https://www.protocols.io/workspaces/george-lab. Detailed protocols for spleen collection, DNA isolation, sample processing, preparing sequencing libraries, and performing the sequencing can be found on protocols.io here: https://www.protocols.io/workspaces/cgord/publications.
Data analysis	The GWAS pipeline can be found here: https://github.com/sanchestm/GWAS-pipeline. All genotype and phenotype data, and GWAS results can be found in the UCSD Library Digital Collections (https://doi.org/10.6075/J0QN675X)

For manuscripts utilizing custom algorithms or software that are central to the research but not yet described in published literature, software must be made available to editors and reviewers. We strongly encourage code deposition in a community repository (e.g. GitHub). See the Nature Portfolio guidelines for submitting code & software for further information.

Data

Policy information about availability of data

All manuscripts must include a data availability statement. This statement should provide the following information, where applicable:

- Accession codes, unique identifiers, or web links for publicly available datasets
- A description of any restrictions on data availability
- For clinical datasets or third party data, please ensure that the statement adheres to our policy

All genotype and phenotype data, and GWAS results can be found in the UCSD Library Digital Collections (https://doi.org/10.6075/J0QN675X). Raw sequence reads

can be found at https://www.ncbi.nlm.nih.gov/bioproject/1022514, accession number PRJNA1022514.

Research involving human participants, their data, or biological material

Policy information about studies with human participants or human data. See also policy information about sex, gender (identity/presentation), and sexual orientation and race, ethnicity and racism.

Reporting on sex and gender	n/a
Reporting on race, ethnicity, or other socially relevant groupings	n/a
Population characteristics	n/a
Recruitment	n/a
Ethics oversight	n/a

Note that full information on the approval of the study protocol must also be provided in the manuscript.

Field-specific reporting

Please select the one below that is the best fit for your research. If you are not sure, read the appropriate sections before making your selection.

☒ Life sciences ☐ Behavioural & social sciences ☐ Ecological, evolutionary & environmental sciences

For a reference copy of the document with all sections, see nature.com/documents/nr-reporting-summary-flat.pdf

Life sciences study design

All studies must disclose on these points even when the disclosure is negative.

Sample size

This study contains data from 20 cohorts of HS rats (approximately 1,125 total experimental rats), spanning over 8 years. The first 17 cohorts were bred at Wake Forest University School of Medicine by Dr. Leah Solberg Woods (NMcwiWFsm:HS #13673907, RRID:RGD_13673907). The last 3 cohorts were bred at the University of California San Diego by Dr. Abraham A. Palmer (McwiWfsmAap:HS #155269102, RRID:RGD_155269102). Rats were shipped or transferred to Dr. Olivier George's lab in batches of 30-60 rats at 3 to 4 weeks of age. RFID chips were inserted into all rats at weaning. The first 6 cohorts were tested at the Scripps Research Institute, and the subsequent 14 cohorts were tested at the University of California San Diego. Each cohort consisted of a similar number of female and male rats.

Data exclusions

Behavioral data, including time stamps for active and inactive lever presses and infusions, was recorded by MED-PC IV software. Experimental notes, including any catheter disconnections, sickness, or death, were also recorded. Of the 1,125 rats that underwent behavioral testing, 158 (13%) were excluded due to sickness, death, catheter failure, or other experimental complications, and 131 (11%) were excluded due to missing or unusable genotype data. Thus, data for part or all of the study was available from 836 rats.

Data from MED-PC files were extracted using custom Python scripts. Data were visually inspected by at least 3 people by plotting all summary data for each day for each individual rat. Missing data from catheter disconnections or computer issues was imputed using Soft-Impute 79. Specifically, we only imputed values for the total number of infusions for short or long access days if the data was missing. We did not impute timestamp data (list of time signatures for infusions or lever presses), or any other behavioral experiment. Imputation used the number of infusions for behavioral data, but did not consider any genetic data. If a rat died during the experiment, data for the days after death were considered missing and were not imputed or used in subsequent behavioral or genetic analysis. In cases of death or sickness, the team visually inspected all behavioral data to determine the likely onset of sickness or cause of death. Only sessions in which the animal appeared healthy were included in the trait analysis. To avoid including data potentially influenced by early signs of illness or experimental complications leading to death, any sessions during the four days immediately preceding death were always excluded, as well as any subsequent sessions after the onset of sickness. All data that passed quality checks, including imputed data, was used in the genetic analysis.

If only a portion of data was kept for a rat, the missing data was denoted with NAs. While we were able to impute total number of infusions on a given day of short or long access, missing timestamp data could not be imputed, thus some calculated traits that rely on timestamp data could not be determined. After applying these quality control measures data from 836 rats were available for the subsequent genetic analysis, however due to missing data that could not be imputed, for some traits, fewer than 836 rats were available for analysis.

Replication

All rats were run through the entire experimental paradigm once to model the progression of addiction-like behaviors. As each individual rat has a unique genetic makeup because the rats are outbred, the rats cannot be replicated and are unique. This reflects human genetic studies and allows us to run a genome-wide association study.

Randomization

We used linear regression to remove the effects of any covariate that explained more than 2% of the variance, including age, cohort, sex, coat color, or weight.

Blinding

Experimenters were blind to genotypes of the rats, and all rats went through the same experimental protocol.

Reporting for specific materials, systems and methods

We require information from authors about some types of materials, experimental systems and methods used in many studies. Here, indicate whether each material, system or method listed is relevant to your study. If you are not sure if a list item applies to your research, read the appropriate section before selecting a response.

Materials & experimental systems

n/a	Involved in the study
x	Antibodies
x	Eukaryotic cell lines
x	Palaeontology and archaeology
	x Animals and other organisms
x	Clinical data
x	Dual use research of concern
x	Plants

Methods

n/a	Involved in the study
x	ChIP-seq
x	Flow cytometry
x	MRI-based neuroimaging

Animals and other research organisms

Policy information about studies involving animals; ARRIVE guidelines recommended for reporting animal research, and Sex and Gender in Research

Laboratory animals	This study contains data from 20 cohorts of HS rats (approximately 1,125 total experimental rats), spanning over 8 years. The first 17 cohorts were bred at Wake Forest University School of Medicine by Dr. Leah Solberg Woods (NMcwiWFsm:HS #13673907, RRID:RGD_13673907). The last 3 cohorts were bred at the University of California San Diego by Dr. Abraham A. Palmer (McwiWfsmAap:HS #155269102, RRID:RGD_155269102).
Wild animals	n/a
Reporting on sex	Each cohort consisted of a similar number of female and male rats. GWAS was run on a total of 836 rats (female = 415, male = 421 rats). We used linear regression to remove the effects of any covariate that explained more than 2% of the variance, including age, cohort, sex, coat color, or weight.
Field-collected samples	n/a
Ethics oversight	All experimental testing was approved by the Institutional Animal Care and Use Committees at the Scripps Research Institute or the University of California San Diego. All rats were treated in compliance with the National Institutes of Health Guide for the Care and Use of Laboratory Animals.

Note that full information on the approval of the study protocol must also be provided in the manuscript.

Plants

Seed stocks	n/a
Novel plant genotypes	n/a
Authentication	n/a
