# Supplementary material for: Genome-wide association study of cocaine self-administration behavior in Heterogeneous Stock rats
Source: Nat Commun. 2026 Jun 11;17:4876. doi: 10.1038/s41467-026-73694-w (PMC13261055; doi:10.1038/s41467-026-73694-w)
Supplement: Supplementary file 14 — Transparent Peer Review file [file 41467_2026_73694_MOESM14_ESM.pdf]

nature portfolio

Peer Review File

Genome-wide association study of cocaine self-administration behavior in Heterogeneous Stock rats

Corresponding Author: Dr Olivier George

This file contains all reviewer reports in order by version, followed by all author rebuttals in order by version.

Version 0:

Reviewer comments:

Reviewer #1

(Remarks to the Author)
This article describes a genomewide association study – GWAS – of a set of traits related to cocaine self-administration in heterogenous stock rats. These rats result from breeding together eight inbred strains over multiple generations and are said to allow for several-megabase mapping resolution (PMID: 27933519). This is by no means comparable to the resolution possible with GWAS mapping in human populations (or presumably even with outbred rats), and it is hard to see how this could be said to allow for individual gene, much less individual variant, fine-mapping. The authors start by describing how cocaine use disorder (CUD) is a major problem but they do not demonstrate how gene mapping in rats might address this problem.

1. It is possible to do GWAS in human populations, i.e. the population of interest, rather than in a model system, so please indicate why it might be more advantageous to work in a model system. One of the advantages of using a model system might be the possibility of generating larger sample sizes than is possible for human studies… but this study is smaller than published human studies.

2. The findings include what are described as six genomewide-significant loci. The significance p-value range should be quoted directly. The p-value to accept genomewide statistical significant is much more lax than that in human studies. The rat genome is nearly the same length as the human genome; if we accept this p-value threshold, this implies that there are orders-of-magnitude fewer independent regions to account for in rats vs. human, with a consequent reduction to the same extent in fine mapping ability. This needs to be discussed as a limitation… presently there is no limitations section in the article at all.

3. Relatedly, fine mapping is hard even in humans, and it is much harder in a higher-linkage disequilibrium system such as the one employed here. Thus, claims to map a risk locus to a specific gene or variant must be carefully defended. In particular the authors claim prominently that one risk region contains genes orthologous to CES1. However, on p8 they indicate that the region also contains other promising candidate loci. It is impossible to identify a causative variant or set of variants based on the information available, so claims to specificity should not be made. There are many such claims in the article and they need to be moderated.

4. A few examples of risk variants that translate between species are quoted, but this cannot be assumed to be the usual situation. A better case needs to be made for how the findings presented here, might relate to human disease risk. The rat traits have inherent interest in themselves but might not relate to human traits.

5. The phenotypes studied are claimed to related to CUD (in humans). The extent to which this might be true is unclear, and the claim needs to be supported more carefully. In the best case though, it seems like some of the phenotypes studied relate to individual CUD criteria, not to CUD, which has a specific diagnostic scheme defined in DSM5 (other schemata are possible but they all tend to be quite similar). Did the authors try to construct a phenotype more closely related to CUD rather than individual criteria?

6. This is a complex GWAS study in a small sample – a total of 836 individuals. The authors should include a replication, if possible.

7. In the introduction, Heterogenous Stock rats are referred to as outbred, therefore capable of greater mapping precision (than inbred?) As noted above, the references I found state that these rats were bred from a small fixed number of inbred strains, and therefore are not outbred. Please clarify and if necessary correct in the article text.

8. The article discusses the use of “SNP genotypes at millions of markers…” This is unnecessarily vague – please state the number of independent markers used.

9. Authors refer to closely-mapped markers in perfect LD (bottom of p10) but considering the LD structure, aren’t all closely-mapped markers necessarily in perfect LD? This is relevant to interpretation of the findings and needs to be addressed rigorously.

Reviewer #2

(Remarks to the Author)
The manuscript by Lara et al. presents the results from a genome-wide association study of voluntary intravenous cocaine self-administration in N/NIH Heterogeneous Stock rats. Sequential phenotypes from short access, long access, progressive ratio, and punishment paradigms are analyzed. Of the twenty-seven phenotypes measured and analyzed, six genome-wide significant loci were identified using permutation-derived thresholds. This is an important study that can significantly advance our understanding of the molecular genetic factors that influence the expression and magnitude of cocaine-reinforced behaviors and (more generally) and support the value of using various genetically diverse rodent models to identify the sources of individual differences in behavior relevant to human disease.

Comments
1. The study examines more than 2 dozen phenotypes, each tested individually with separate genome-wide using permutation-derived thresholds. While per-trait thresholds are common practice in GWAS, the relatively large number of traits here raises the possibility of inflated false positives across the phenome. The main results are compelling, but it would be helpful for the authors to include a brief statement clarifying their rationale for using trait-wise thresholds and to provide a limited sensitivity analysis. For example, applying a simple correction based on the effective number of independent traits, or reporting false discovery rates across traits for the lead loci, would reassure readers that the associations are robust without requiring a fundamentally different analytic framework. On a related note, the first two paragraphs of the Results focus on an understanding of the way that subsets of these measures load on one another (as would be expected), yet these analyses and the results of the dimension reduction efforts are not carried forward into the GWAS. Some comment on this is recommended.

2. There seems to be no discussion on how sample sizes were selected. Was a priori power analysis performed?

3. There is little information to indicate how certain follow-up analyses were done. More specific information on how coding/expression variants and tissue-specific eQTL were performed and determined is needed.

4. Moreover, a significant amount of procedural detail is not included in this manuscript or in the supplemental files. Instead, links to open access methods are provided, but those methods are not detailed. In just one example, neither the source of the cocaine is provided, nor is information on whether the unit dose administered was calculated as the sale or the base.

5. Were any analyses conducted to calculate sex effects either at the phenotype level or for GWAS?

6. The manuscript notes that inactive lever presses were recorded but had no consequence (“programmed” consequence, that is). However, no analyses of inactive lever responding or lever discrimination are presented. Given that discrimination between active and inactive levers is an important control for specificity of responding, please either include a summary of these measures (e.g., descriptive statistics across phases) or clarify why they were not analyzed further.

7. The manuscript states that total infusions were imputed for missing sessions using SoftImpute, but it is unclear how extensive this imputation was. To evaluate the potential impact on escalation and infusion count measures, please provide descriptive statistics on the number of imputed values. Specifically:
• How many data points were imputed in total across all traits?
• What proportion of sessions were imputed within each phenotype?
• How were imputations distributed across animals (e.g., mean, median, and range of imputed sessions per subject)?
This information would allow readers to judge whether the imputations were sparse and evenly distributed or whether certain animals relied heavily on imputed values, which could influence the robustness of the results.

7. Catheter patency was determined by brevital test after the LgA sessions. Was this performed on an active testing day; that is, before or after active testing? For those rats that failed the test, the investigators inspected the data and used poorly-defined subjective evaluation to determine when to start excluding data. The justification for this is weak and raises questions about rigor. If certain genotypes were more commonly associated with catheter patency loss (a not unlikely possibility) or with greater behavioral variability (also, a likely possibility), this could alter the results of the GWAS. Some comment on this would be helpful. Moreover, why wasn’t catheter patency tested again after the conclusion of the entire phenotyping pipeline?

8. A table with key definitions of terms and acronyms in the main text would be helpful. Note, there seem to be acronyms with

no definitions, e.g., “LgA Late PII” with no definition for PII.

9. A table reporting the sig. GWAS hits with key information in the main text would be helpful to readers.

Reviewer #3

(Remarks to the Author)
I co-reviewed this manuscript with one of the reviewers who provided the listed reports. This is part of the Nature Communications initiative to facilitate training in peer review and to provide appropriate recognition for Early Career Researchers who co-review manuscripts.

Reviewer #4

(Remarks to the Author)
I greatly enjoyed this concise and informative paper, which efficiently conveys a large amount of underlying data and analysis. The in-depth phenotyping and sophisticated bioinformatics provide an attractive package and provides useful insights into cocaine use disorder (CUD). Further, the identification of the Ces genes as candidates for CUD has therapeutic implications. The supplementary data in .csv and .html format was helpful in understanding the results.

The following suggestions are all minor, but may be helpful.

Minor comments
1. Figure 1C legend states “non-significant values shown in white”, but key indicates Spearman’s correlation coefficient (not significance value). Might be worthwhile including a parallel key showing -log10P values.
2. Figure 1B. Might be worth referencing M=male and F=female in legend.
3. While reading, a large number of questions arose concerning the nature of the phenotypes. For example, Figure 1C: Under LgA Escalation cluster, both “Total Infusions” and “LgA Total Infusions” appear. I wondered what the difference between them is? (Is “Total Infusions” == “LgA Total Infusions” + “ShA Total Infusions”.)

In Figure 1F: What is “LgA Early PII” and “LgA Late PII”? (Presumably, post infusion interval, and for “LgA Early TO” and “LgA Late TO” is “timeout”?) Do ShA and LgA early and late sessions correspond to an average of the first and last 3 sessions respectively (Figure 1A suggests this interpretation)? Or the initial and final sessions?

How are “late loading” “early loading” defined?

In fact, I see that many of these terms and others are clearly defined in Supplementary Table 1 and in the Methods. Unfortunately, due to Journal formatting, the Methods section appears at the end of the manuscript. Mentioning where these and other terms are defined earlier in the Results section of the main manuscript and pointing the reader both to the resources in Supplementary Table 1 and the Methods may help assuage distracting questions in the mind of the reader.

4. Figure 2B. “the color reflects the behavioral cluster from which the trait belongs (Figure1B)”. Should “Figure 1B” be “Figure 1C”?
5. Do any of the 3 missense mutations in the Ces cluster map near known active sites of the enzymes or have strong phyloP/Gnomad scores?
6. Is the term “post infusion interval” a bit confusing? Perhaps simply “infusion interval” is easier to understand.
7. The Rasd2 eQTL is presumably a cis-eQTL (rather than trans) and the R2=1 refers to the linkage disequilibrium rather than the correlation of the SNP with the expression of the gene?
8. A brief title/description in the .csv files (one entry?) of the supplementary tables would be helpful for rapid understanding.
9. Methods. I was curious about the missing data as a result of catheter disconnections or computer malfunction. It would be interesting to include the number of data loss instances, the number of rats affected by these issues and how much data was lost (and hence imputed) for each instance.

Version 1:

Reviewer comments:

Reviewer #1

(Remarks to the Author)
I appreciate the conscientious response to my earlier critique, but that response has confirmed my reservations about this study and its relevance.

1. Earlier critique and response included the following: “This is by no means comparable to the resolution possible with GWAS mapping in human populations (or presumably even with outbred rats)…” Response: We agree with the reviewer that

humans are better for fine mapping. However, there is a trade off … the greater linkage disequilibrium (LD) observed in HS rats reduces the effective number of hypotheses tested, greatly increasing power, at the expense of mapping precision. This is a key reason that we are able to obtain genome-wide significant results with a sample size that would be underpowered in humans.

Because of this decreased resolution, this study is not comparable to a GWAS in humans. As such the title “Genome-wide association study of cocaine self-administration 1 behavior…” does not carry the expected meaning to an investigator familiar with this kind of analyses in humans. A more accurate title would be, “Low-resolution genomewide association study…” As the authors acknowledge, this is one of the costs of accomplishing the work and obtaining significant results in a small sample. The loss of resolution is a critical problem and, as the authors confirm elsewhere, compromises identification of specific genes as opposed to relatively broad regions. Ces1 is identified “as a prioritized candidate” and mentioned in the abstract but it is mapped to a region that contains other loci and is not supported with sufficient evidence to be highlighted so strongly. Similarly the reference to “perfect linkage disequilibrium” is not meaningful because the high levels of LD overall most likely make this the most usual circumstance. I asked the authors to address this question in the previous critique but they did not (see item 4 below). Any reference to “perfect linkage disequilibrium” should be tempered by a consideration of the overall LD in this model.

2. The authors refer to the rodent behavior as “CUD-like”. This is not sufficiently supported. It would be more accurate to refer to it as a cocaine consumption behavior – or in some other way that does not draw an explicit parallel to a human diagnosis that the data do not support fully.

3. The lack of a replication for this finding in a small sample remains a serious limitation.

4. Item 9 from the first critique “Authors refer to closely-mapped markers in perfect LD (bottom of p10) but considering the LD structure, aren’t all closely-mapped markers necessarily in perfect LD? This is relevant to interpretation of the findings and needs to be addressed rigorously” is not
responded seriously. This requires a rigorous response.

Reviewer #2

(Remarks to the Author)
The responses provided and edits performed were generally clear and responsive to the questions raised in the initial review. There is one point to raise at this stage:

In response to the initial review, the authors added text on lines 73-75 that "exposure ... to addictive drugs" would be "unethical in humans". As written, this is incorrect. There are a number of clinical or research reasons why naive people may be exposed to a potentially addictive drug in an ethical manner. This sentence should be substantially revised.

Reviewer #3

(Remarks to the Author)
I co-reviewed this manuscript with one of the reviewers who provided the listed reports. This is part of the Nature Communications initiative to facilitate training in peer review and to provide appropriate recognition for Early Career Researchers who co-review manuscripts.

Reviewer #4

(Remarks to the Author)
The authors have fully answered my questions.

Open Access This Peer Review File is licensed under a Creative Commons Attribution 4.0 International License, which permits use, sharing, adaptation, distribution and reproduction in any medium or format, as long as you give appropriate credit to the original author(s) and the source, provide a link to the Creative Commons license, and indicate if changes were made.
In cases where reviewers are anonymous, credit should be given to 'Anonymous Referee' and the source.
The images or other third party material in this Peer Review File are included in the article’s Creative Commons license, unless indicated otherwise in a credit line to the material. If material is not included in the article’s Creative Commons license and your intended use is not permitted by statutory regulation or exceeds the permitted use, you will need to obtain permission directly from the copyright holder.
To view a copy of this license, visit https://creativecommons.org/licenses/by/4.0/

Dear Reviewers,

We appreciate the thoughtful comments from the reviewers and thank them for their time and attention. We have carefully addressed every point raised in the critique. Most notably, we have expanded the Introduction to better articulate the unique advantages and mapping resolution of the HS rat model and added a comprehensive 'Limitations' section to the Discussion to transparently address significance thresholds, multiple testing, and cross-species translation. Furthermore, to demonstrate the rigor of our phenotyping pipeline, we have included new supplemental data quantifying lever discrimination (Supplemental Figure 1) and data imputation rates (Supplemental Table 8), alongside clarified methods for catheter patency and genetic follow-up. We believe this rigorous review process has significantly improved the clarity and precision of the manuscript, ultimately strengthening our initial conclusions. Responses to comments are below in **green** and additional manuscript text is designated in **orange** with manuscript line numbers provided.

Reviewer #1 (Remarks to the Author):

This article describes a genomewide association study – GWAS – of a set of traits related to cocaine self-administration in heterogenous stock rats. These rats result from breeding together eight inbred strains over multiple generations and are said to allow for several-megabase mapping resolution (PMID: 27933519).
We thank the reviewer for raising this point and giving us the opportunity to clarify mapping resolution. While the paper cited by the reviewer reports that the HS rats enable mapping resolution on the order of several megabases, we believe this report to be outdated. In our own experience, it is common to map loci to <1Mb regions, for example, Figure 2C, which identifies a region spanning from about 13.85 to 14.05 Mb on rat Chromosome 19 (~200 Kb).

This is by no means comparable to the resolution possible with GWAS mapping in human populations (or presumably even with outbred rats),
We agree with the reviewer that humans are better for fine mapping. However, there is a trade off -- as we have discussed (e.g., PMID 31228160), the greater linkage disequilibrium (LD) observed in HS rats reduces the effective number of hypotheses tested, greatly increasing power, at the expense of mapping precision. This is a key reason that we are able to obtain genome-wide significant results with a sample size that would be under-powered in humans.

and it is hard to see how this could be said to allow for individual gene, much less individual variant, fine-mapping.

We do not claim to fine map to an individual gene or variant. However, we highlight several genes that are 1) within the regions identified (Figure 2C) and that have either coding variants or eQTLs that are in LD with the causal variants. Unlike humans, where fine mapping is the only available tool, rats are amenable to experimental genetic and pharmacological manipulations, so the implicated genes and variants can be directly tested in future studies.

The authors start by describing how cocaine use disorder (CUD) is a major problem but they do not demonstrate how gene mapping in rats might address this problem.
We agree with the reviewer that this was a major omission. In the introduction, we now highlight key advantages of performing genetic studies in rodents.

Manuscript Introduction edit (lines 70-87):

> Rodent models provide a complementary resource for exploring the genetic basis of CUD-like behaviors under tightly controlled experimental conditions. There are several advantages to using rodent models. First, the greater linkage disequilibrium (LD) found in rodents provides greater power albeit at the expense of mapping precision (PMID 31228160). In addition, rodent models allow exposure of naïve animals to addictive drugs, which would be unethical in humans. Controlled drug exposure allows examination of precise and objective drug-taking patterns (e.g. the post infusion intervals of drug self-administration, measures of demand (e.g. through behavior economics procedures such at the progressive ratio paradigms used in this study). Furthermore, rodents are exposed to uniform environmental conditions prior to drug exposure and avoid the effects of genetically influenced environmental exposures (e.g. socioeconomic factors, exposure to trauma due to risk-taking behaviors, and nature/nurture effects). Finally, rodents offer the possibility of experimental manipulations, either using experimental therapeutic agents for which safety has not yet been established, and by genetic manipulations using CRISPR, viral vectors and opto- and chemo-genetic manipulations. Thus, the cellular and circuit-level effects of identified polymorphisms can be experimentally investigated, which is advantageous for understanding the mechanism and development of therapeutic agents. For these reasons, we and others have pursued rodent studies of CUD-like behaviors.

1. It is possible to do GWAS in human populations, i.e. the population of interest, rather than in a model system, so please indicate why it might be more advantageous to work in a model system. One of the advantages of using a model system might be the possibility of generating larger sample sizes than is possible for human studies... but this study is smaller than published human studies.
We thank the reviewer for encouraging us to add a rationale for doing genetic studies in rats. We specifically write that rodent studies are *complementary*, and we do not propose them as a replacement for human GWAS. The reviewer mentions sample size, which is a proxy for power. As we mentioned above, the greater LD in HS rats trades mapping

precision for power, meaning that studies in HS rats do not need to have larger sample sizes to provide comparable or even greater power. In response to this and a prior comment by this reviewer (see above), we added a section to the introduction explaining some of the advantages of doing these studies in rats (lines 70-87). We would also direct this reviewer to the paragraph on lines 96-103, which provide additional justification/advantages of doing this work in rats versus humans.

2. The findings include what are described as six genomewide-significant loci. The significance p-value range should be quoted directly. The p-value to accept genomewide statistical significant is much more lax than that in human studies. The rat genome is nearly the same length as the human genome; if we accept this p-value threshold, this implies that there are orders-of-magnitude fewer independent regions to account for in rats vs. human, with a consequent reduction to the same extent in fine mapping ability. This needs to be discussed as a limitation...

This is related to a point we made above -- the longer range LD in HS rats means that there are fewer independent hypotheses tested, so the threshold for significance is expected to be less stringent. Unlike human studies, which use a threshold of 5×10^{-8} (suggested almost 30 years ago based on $0.05/10^{6}$; PMID 8801636), model organism studies typically use permutation because each population has different LD patterns, so it is not possible to use a 'one-size fits all' threshold. This is now explicitly addressed in the text we added to the introduction (see above). However, we agree with the reviewer that it is better to be forthcoming about the significance threshold. Therefore, we have edited the relevant sentence in the abstract.

Abstract edit (lines 39-40):

> We identified six genome-wide significant associations ($>-\log_{10}(p)=5.58$; $\alpha = 0.05$ by permutation).

...presently there is no limitations section in the article at all.

We agree that there should be an explicit limitations paragraph; therefore, we have now added one at the end of the discussion section.

Manuscript Discussion edit (lines 426-449):

> Limitations: First, we use significant thresholds that differ from human GWAS. Genome-wide significance thresholds were derived using permutation testing to empirically control the rate of false positives ($\alpha = 0.05$) based on the LD structure of the HS rat population. HS rats have more extensive LD and fewer independent markers than humans. This results in less stringent p-value thresholds than the conventional human GWAS threshold (5×10^{-8}). This reflects a tradeoff between statistical power and mapping resolution. While this limits our ability to pinpoint a single causal variant, we are encouraged by the significant findings in this study that reveal shorter LD blocks with few, but biologically relevant genes within the interval. Although permutation thresholds appropriately control false positives for the HS rat population, they do not adjust for the total number of phenotypes examined.

Second, we did not measure cocaine metabolism directly, and the association with *Ces1* suggests that pharmacokinetic variation may contribute to the observed behavioral differences. Third, our self-administration behaviors are restricted to intravenous self-administration. While this is a well-established model of drug taking, other routes of administration used by humans are not captured. Furthermore, future studies that incorporate alternative choices such as food or social rewards may yield different outcomes. Fourth, sex was included as a covariate to account for baseline differences between males and females, but the current study is underpowered to identify sex x genotype interactions. Finally, the existence of a coding variant in HS rats may not directly translate to a human risk variant. However, convergent evidence across genes and molecular pathways can provide insight into the conserved biological mechanisms of addiction. The present findings serve as a basis for future follow up and cross-species studies.

3. Relatedly, fine mapping is hard even in humans, and it is much harder in a higher-linkage disequilibrium system such as the one employed here. Thus, claims to map a risk locus to a specific gene or variant must be carefully defended. In particular the authors claim prominently that one risk region contains genes orthologous to CES1. However, on p8 they indicate that the region also contains other promising candidate loci. It is impossible to identify a causative variant or set of variants based on the information available, so claims to specificity should not be made. There are many such claims in the article and they need to be moderated.
We agree that causal variants need to be adequately defended; thus, we are careful not make overly specific claims, but rather present evidence to prioritize the genes within the significant loci. We have carefully reviewed the manuscript to ensure that we do not make causal claims, and that all the sections consistently frame these regions as "containing multiple candidate genes", with *Ces1* highlighted as a prioritized candidate.

4. A few examples of risk variants that translate between species are quoted, but this cannot be assumed to be the usual situation. A better case needs to be made for how the findings presented here, might relate to human disease risk. The rat traits have inherent interest in themselves but might not relate to human traits.
We agree that the cross-species translation of specific risk variants cannot be assumed. This study is not intended to resolve the alignment of specific risk variants, but rather identify genetic loci associated with well-defined cocaine self-administration behaviors under controlled conditions. While the direct correspondence between rat and human variants is unlikely, these loci can inform human disease risk at the gene and pathway level by prioritizing candidate genes using human GWAS results, gene expression data, and known functional variation. We have revised the discussion limitation section to clarify that the findings are most informative at the broader gene level, and that integrative cross-species analyses should be done for future studies (lines 445-449, see comment #2 above).

5. The phenotypes studied are claimed to related to CUD (in humans). The extent to which this might be true is unclear, and the claim needs to be supported more carefully. In the best case though, it seems like some of the phenotypes studied relate to individual CUD criteria, not to CUD, which has a specific diagnostic scheme defined in DSM5 (other schemata are possible but they all tend to be quite similar). Did the authors try to construct a phenotype more closely related to CUD rather than individual criteria?
The benefit of using this behavioral paradigm is that we can dissect the genetic contribution to individual aspects of cocaine self-administration behavior as well as overall patterns of behavior related to addiction. We want to clarify that we never claim these rats have CUD, but rather we are using HS rats to model CUD-like behavioral features under controlled experimental conditions. We use a paradigm of extended access combined with measures relevant to compulsive drug seeking and taking using progressive ratio responding and responding despite adverse consequences. We chose this model because of its relevance to CUD. It has been widely adopted because it has been shown to produce escalation of intake and compulsive drug seeking that correspond to multiple DSM-5 criteria, including tolerance, withdrawal, and continued use despite negative consequences. One of the advantages of this paradigm is that we can individually analyze these distinct components of drug self-administration, especially the variation over time in self-administration behavior across individuals. In addition to the individual behaviors, we have used the first principal component of the self-administration measures as a composite phenotype, which captures a large proportion of the variance across behaviors (Figure 1D-F). While there are other dimension reduction methods, we used principal component analysis because we wanted to have a continuous measure, which could be used as a trait for GWAS that would capture the variance across the correlated traits while maintaining individual-level variation.

6. This is a complex GWAS study in a small sample – a total of 836 individuals. The authors should include a replication, if possible.
Unfortunately, there are no similar datasets available for replication, and it took about 8 years to collect the data. The importance of this study is that it is the first attempt to genetically characterize cocaine self-administration in a large cohort of outbred rats. This study has the benefit of being highly controlled and deeply comprehensive, and such a large undertaking is infeasible given time and other constraints. The combination of a deep phenotyping pipeline and a large cohort of a genetically heterogeneous model system is a unique dataset, and we intend to continue using this valuable dataset for future studies to fully realize its potential. We have also taken steps to ensure the robustness of our findings despite not having an independent replication. We use permutation testing, as described in the methods and question #2, to set genome-wide significance thresholds, which is a widely used approach to control for error. Additionally, we have implemented an extremely stringent quality control of phenotypes and genotypes as described in the methods, which helps de-noise any downstream analysis.

7. In the introduction, Heterogenous Stock rats are referred to as outbred, therefore capable of greater mapping precision (than inbred?) As noted above, the references I found

state that these rats were bred from a small fixed number of inbred strains, and therefore are not outbred. Please clarify and if necessary correct in the article text.
The HS rat population is indeed derived from an original fixed set of inbred founder strains, but has since been maintained through over 100 generations of outbreeding. This outbreeding maximizes genetic heterogeneity through a mating design that minimizes breeding between closely related individuals from the same families (as opposed to inbreeding, which fixes the genetic makeup of the offspring). After so many generations of outbreeding, the resulting individual rats are distinct genetic mosaics of the founder haplotypes which have been recombined. The reference given (PMID 27933519) describes this outbreeding for the HS rat and mouse population, and is consistent with how we describe the population. The references we cite, one of which includes the first author of the reference given, also describe this process of maintaining an outbred population for modeling genetic heterogeneity (PMID 31228160). We have clarified in the introduction that this outbreeding enables greater mapping precision compared to traditional intercrossing or backcrossing mapping strategies, which have much larger LD blocks.

Manuscript Introduction edit (lines 96-99):

> Outbred populations such as the Heterogeneous Stock (HS) rats provide a high degree of genetic heterogeneity and lower levels of linkage disequilibrium, enabling greater mapping precision than inbred or traditional F_2 intercross populations.

8. The article discusses the use of “SNP genotypes at millions of markers…” This is unnecessarily vague – please state the number of independent markers used.
We have edited the introduction to include “5,446,333 SNPs” (lines 114-115) and this information can also be found in the methods under “Genotyping”.

9. Authors refer to closely-mapped markers in perfect LD (bottom of p10) but considering the LD structure, aren’t all closely-mapped markers necessarily in perfect LD? This is relevant to interpretation of the findings and needs to be addressed rigorously.
In an F_2 cross between two inbred strains, all closely mapped markers are indeed in LD. However, in the HS rat population, there are eight founder haplotypes. Thus, adjacent markers may have different LD relationships because they have different strain distribution patterns across the eight founders, as in other populations (F_2 crosses and outbred humans).

Reviewer #2 (Remarks to the Author):
The manuscript by Lara et al. presents the results from a genome-wide association study of voluntary intravenous cocaine self-administration in N/NIH Heterogeneous Stock rats. Sequential phenotypes from short access, long access, progressive ratio, and punishment paradigms are analyzed. Of the twenty-seven phenotypes measured and analyzed, six genome-wide significant loci were identified using permutation-derived thresholds. This is an important study that can significantly advance our understanding of the molecular genetic factors that influence the expression and magnitude of cocaine-reinforced

behaviors and (more generally) and support the value of using various genetically diverse rodent models to identify the sources of individual differences in behavior relevant to human disease.
We appreciate the generous feedback and agree that this works reinforces the value of rodent modeling as a complementary method in neuropsychiatric genetics.

Comments
1. The study examines more than 2 dozen phenotypes, each tested individually with separate genome-wide using permutation-derived thresholds. While per-trait thresholds are common practice in GWAS, the relatively large number of traits here raises the possibility of inflated false positives across the phenome. The main results are compelling, but it would be helpful for the authors to include a brief statement clarifying their rationale for using trait-wise thresholds and to provide a limited sensitivity analysis. For example, applying a simple correction based on the effective number of independent traits, or reporting false discovery rates across traits for the lead loci, would reassure readers that the associations are robust without requiring a fundamentally different analytic framework.
We agree that testing multiple phenotypes raises the possibility of inflated false positives across traits. In our experience, using FDR increases the number of associations, but this is not a conventional approach for GWAS and the results are not as easily compared across studies. Using a correction for the effective number of tests is also not simple, since both the nearby SNPs and the traits are correlated (see Figure 1C). To address this comment, we have amended the discussion section (limitations paragraph lines 426-449) to highlight this issue. (This change also addresses reviewer #1 question 2).

On a related note, the first two paragraphs of the Results focus on an understanding of the way that subsets of these measures load on one another (as would be expected), yet these analyses and the results of the dimension reduction efforts are not carried forward into the GWAS. Some comment on this is recommended.
We appreciate this comment, which made us realize that the text needed to be revised to clarify our use of principal components. We did use the first principal component of the long access behaviors as a trait for GWAS. The trait is first described in the result section “principal component analysis of cocaine self-administration,” (see Figure 1D-F). We then show the results of GWAS for this trait in Figure 2A (there is a genome-wide significant association for PC1 on Chromosome 19). Finally, in Figure 2B we show that this principal component is highly heritable, emphasizing the value of dimension reduction to capture a meaningful biological signal.

While not in the main paper figures, the Supplemental Report shows shared underlying genetic findings across traits with the Manhattan plots and tables. Specifically, the individual long access traits have subthreshold signals at several loci that overlap with those identified in other traits as well as the summary PC1 measure. For example, total intake and early loading have subthreshold signals that overlap with the significant loci for PC1 on Chromosome 19. We have added a sentence in the Results section “principal

component analysis of cocaine self-administration," clearly stating that PC1 was used in the GWAS (lines 165-166).

2. There seems to be no discussion on how sample sizes were selected. Was a priori power analysis performed?

When applying for funding we had proposed to phenotype 1,000 HS rats, for which we had performed a power analysis. The sample size was also informed by our prior experience with similar behavioral phenotypes that we have studied in HS rats (e.g. PMID 40000848). We tested a total of 1,125 rats, however 158 were lost for technical reasons (sickness, death, catheter failure, or other experimental complications) and an additional 131 were lost because genotype data was unavailable.

3. There is little information to indicate how certain follow-up analyses were done. More specific information on how coding/expression variants and tissue-specific eQTL were performed and determined is needed.

We agree and have amended the Method "Genetic Analysis" section.

Manuscript Methods edits (lines 666-671):

> Within each significant locus, we focused on cis-expression quantitative trait loci (eQTL) and splice QTLs (sQTLs), which were taken from RatGTEx.org, as well as coding variants that were in LD ($r^2 > 0.6$) with the peak SNP. If known eQTLs or sQTLs were present, we prioritized changes in brain regions that were relevant to each trait. Coding variants within each locus were scored using the Ensembl Variant Effect Predictor (VEP).

4. Moreover, a significant amount of procedural detail is not included in this manuscript or in the supplemental files. Instead, links to open access methods are provided, but those methods are not detailed. In just one example, neither the source of the cocaine is provided, nor is information on whether the unit dose administered was calculated as the sale or the base.

We regret the confusion and would like to point to our method section at the end of the manuscript (as per the instructions to authors for Nature Communications). The cocaine administration information, as well as all other drug information can be found on lines 498-504, 513 - 515. Additionally, while we have provided extensive details in this paper, one of our previous papers provides additional methodological details (PMID 39484794). We have also amended the methods in several places (see comments below).

5. Were any analyses conducted to calculate sex effects either at the phenotype level or for GWAS?

We agree that sex effects are an important and interesting line of inquiry. Our previous behavior-focused paper discusses sex effects at the phenotype level (PMID 39484794). There are sex differences in some behaviors, most notably with females self-administering more cocaine compared to males, which is consistently observed in our expanded dataset. This general trend can be seen in Figure 1B with the pink and blue bar on the side

denoting females (F) and males (M). However, the present study did not have sufficient statistical power to find sex x genotype interactions, and such an analysis would have increased by 3-fold the number of tests performed (relevant to the first comment by this reviewer). Instead of performing a sex-specific GWAS, we regressed out the effect of sex (see "Trait Processing" in the Methods section). In response to this comment, as well as a similar comment by reviewer #1, we have now mentioned this issue in the Limitations paragraph in the Discussion section (lines 426-449).

6. The manuscript notes that inactive lever presses were recorded but had no consequence ("programmed" consequence, that is). However, no analyses of inactive lever responding or lever discrimination are presented. Given that discrimination between active and inactive levers is an important control for specificity of responding, please either include a summary of these measures (e.g., descriptive statistics across phases) or clarify why they were not analyzed further.
This is an excellent point. In our prior paper (PMID 39484794) we showed that rats start differentiating the active from the inactive levers on the first day of ShA1 with a >3/1 ratio between active/inactive pressing by ShA7 (78% vs 22% +/- 1.3, $p<0.001$, see Supplemental Figure 1 from PMID 39484794). To address this comment, we have now included a Supplemental Figure 1 that shows the average number of active versus inactive lever presses across sessions, as well as the percent of active or inactive presses out of the total lever presses. Inactive lever pressing is significantly lower by the end of the 14 days of long access. Because the rats successfully distinguish between the two levers, the variable number of infusions across rats is not a result of acquisition issues. We also now reference this finding in the methods section in this paper (lines 519-523).

7. The manuscript states that total infusions were imputed for missing sessions using SoftImpute, but it is unclear how extensive this imputation was. To evaluate the potential impact on escalation and infusion count measures, please provide descriptive statistics on the number of imputed values. Specifically:
• How many data points were imputed in total across all traits?
• What proportion of sessions were imputed within each phenotype?
• How were imputations distributed across animals (e.g., mean, median, and range of imputed sessions per subject)?
This information would allow readers to judge whether the imputations were sparse and evenly distributed or whether certain animals relied heavily on imputed values, which could influence the robustness of the results.
Specific details about imputation can be found in the "Phenotype data processing" section of the Methods (lines 570 – 592). Less than 1% of all individual rat data was imputed across all short or long access sessions. Additionally we have added a Supplemental Table 8 that outlines which values were imputed per rat across the short and long access sessions.

8. Catheter patency was determined by brevital test after the LgA sessions. Was this performed on an active testing day; that is, before or after active testing? For those rats that failed the test, the investigators inspected the data and used poorly-defined

subjective evaluation to determine when to start excluding data. The justification for this is weak and raises questions about rigor. If certain genotypes were more commonly associated with catheter patency loss (a not unlikely possibility) or with greater behavioral variability (also, a likely possibility), this could alter the results of the GWAS. Some comment on this would be helpful. Moreover, why wasn't catheter patency tested again after the conclusion of the entire phenotyping pipeline?
Catheter patency is indeed a critical assessment for intravenous self-administration studies. To clarify, catheter patency was tested three times: immediately after surgery, immediately after the last short access session, and immediately after the final long access session. This allows us to more precisely define the time when the catheter failure occurred. As this was conducted right after active testing, this directly addresses the reviewer's concern about timing. If the catheter was patent after short access but not after long access, the data from the short access was retained. Additionally, catheter failure produces well-defined and reproducible behaviors that allow for further identification of the likely day of failure, including 1) burst responding during the session or at the beginning of the first session following loss of patency, and 2) a sustained decrease in self-administration relative to previous sessions. When these criteria were clearly observable before a brevital test failure, behavioral data was excluded for any session that was obviously affected by catheter issues. When these were not clearly observable and there was a brevital test failure, all data was excluded. These criteria were applied uniformly and independent of genotype. Finally, unlike experiments that compare two or more groups (e.g. treatment vs. control), a GWAS examines millions of SNPs. Since we are not aware of those millions of SNP genotypes when making these decisions, we do not believe that we could introduce a systematic bias that would lead to false positive associations. While the loss of individuals due to catheter failure may reduce power by introducing additional noise, retaining data from rats that were no longer reliably receiving drug would be more problematic.

Method clarification (lines 530-541):

> Brevital test was performed three times, after surgery, immediately after the last session of ShA, and immediately after the last session of LgA, in order to test catheter patency…Exclusion decisions were made by individuals who were blind to SNP genotypes, ensuring bias was not introduced to the phenotype data for the sample.

9. A table with key definitions of terms and acronyms in the main text would be helpful. Note, there seem to be acronyms with no definitions, e.g., "LgA Late PII" with no definition for PII.
We apologize for the oversight and have reviewed the paper in its entirety to ensure clarity for any acronyms (also see Figure legends). We have also added a table of terms and taken out any acronyms that are better understood as their full name.

Manuscript edits:

> Figure 1 legend: (Acronyms: ShA: short access, PR: progressive ratio, LgA: long access, TO: timeout, PII: post infusion interval, F: female, M: male)

Figure 2 legend: (Acronyms: ShA: short access, PR: progressive ratio, LgA: long access)
Table 1. Terms and acronyms

10. A table reporting the sig. GWAS hits with key information in the main text would be helpful to readers.
Figure 2A provides this information in graphical form. We also provide much more detailed tables in Supplemental Tables 4-7 that have extensive information (peak SNP, MAF, effect size, p-value, genes in the interval, eQTLs, sQTLs, and coding SNPs). Because these tables are so large they were too big to put in the main paper.

Reviewer #3 (Remarks to the Author):

I co-reviewed this manuscript with one of the reviewers who provided the listed reports. This is part of the Nature Communications initiative to facilitate training in peer review and to provide appropriate recognition for Early Career Researchers who co-review manuscripts.

Reviewer #4 (Remarks to the Author):

I greatly enjoyed this concise and informative paper, which efficiently conveys a large amount of underlying data and analysis. The in-depth phenotyping and sophisticated bioinformatics provide an attractive package and provides useful insights into cocaine use disorder (CUD). Further, the identification of the Ces genes as candidates for CUD has therapeutic implications. The supplementary data in .csv and .html format was helpful in understanding the results.

The following suggestions are all minor, but may be helpful.
We are very grateful to the reviewers for their kind comments and have addressed the concerns and implemented several of their suggestions.

Minor comments
1. Figure 1C legend states “non-significant values shown in white”, but key indicates Spearman’s correlation coefficient (not significance value). Might be worthwhile including a parallel key showing -log10P values.
We appreciate that there are a lot of values to interpret in the graph. Color (diverging red to blue) indicates the strength and direction of the relationship, while a completely empty cell indicates the relationship was not significant. To be clearer and provide the data, we have included an additional Supplemental Table 2 with p-values of the correlations.

2. Figure 1B. Might be worth referencing M=male and F=female in legend.
We have revised the figure legend to include M and F.

3. While reading, a large number of questions arose concerning the nature of the phenotypes. For example, Figure 1C: Under LgA Escalation cluster, both “Total Infusions” and “LgA Total Infusions” appear. I wondered what the difference between them is? (Is “Total Infusions” == “LgA Total Infusions” + “ShA Total Infusions”.)
In Figure 1F: What is “LgA Early PII” and “LgA Late PII”? (Presumably, post infusion interval, and for “LgA Early TO” and “LgA Late TO” is “timeout”?) Do ShA and LgA early and late sessions correspond to an average of the first and last 3 sessions respectively (Figure 1A suggests this interpretation)? Or the initial and final sessions?
How are “late loading” “early loading” defined?
In fact, I see that many of these terms and others are clearly defined in Supplementary Table 1 and in the Methods. Unfortunately, due to Journal formatting, the Methods section appears at the end of the manuscript. Mentioning where these and other terms are defined earlier in the Results section of the main manuscript and pointing the reader both to the resources in Supplementary Table 1 and the Methods may help assuage distracting questions in the mind of the reader.
We agree that all the terms should be clarified and easier to read. We have added a table of terms and ensured that all acronyms have been defined (Table 1). Additionally, we would like to point to the Methods section “Addiction-like trait calculations” for more detailed descriptions of calculations and definitions. For both short access and long access, “early” and “late” denote if a trait was derived from the first 3 or last 3 or 4 sessions, respectively (lines 595-597).

4. Figure 2B. “the color reflects the behavioral cluster from which the trait belongs (Figure1B)”. Should “Figure 1B” be “Figure 1C”?
Yes, thank you for catching this error. This has been fixed in the figure legend (lines 260).

5. Do any of the 3 missense mutations in the Ces cluster map near known active sites of the enzymes or have strong phyloP/Gnomad scores?
We are currently performing experiments to assess the effects of these variants; however, these studies will likely take another year or more to complete. Therefore, we plan to leave this question for a future publication.

6. Is the term “post infusion interval” a bit confusing? Perhaps simply “infusion interval” is easier to understand.
After significant deliberation with our colleagues, we chose post infusion interval to signify that the trait is related to behavior occurring after drug delivery, and we respectively would prefer to keep this name.

7. The Rasd2 eQTL is presumably a cis-eQTL (rather than trans) and the R2=1 refers to the linkage disequilibrium rather than the correlation of the SNP with the expression of the gene?
Yes, all reported QTLs are cis and we have clarified this in the methods (see comment #3 from reviewer #2, and lines 666-671). The r^2 value refers to the LD between the top SNP for

the eQTL and the top SNP for the phenotypes, not the expression of the gene (gene expression was not measured in any of the rats in this study).

8. A brief title/description in the .csv files (one entry?) of the supplementary tables would be helpful for rapid understanding.
We have added a table detailing all the terms and files (Table 1).

9. Methods. I was curious about the missing data as a result of catheter disconnections or computer malfunction. It would be interesting to include the number of data loss instances, the number of rats affected by these issues and how much data was lost (and hence imputed) for each instance.
We have now addressed this issue more systematically (also see our responses to Reviewer #2 comment #7: Specific details about imputation can be found in the "Phenotype data processing" section of the Methods (lines 570 – 592). Less than 1% of all individual rat data was imputed across all short or long access sessions. Additionally we have added a Supplemental Table 8 that outlines which values were imputed per rat across the short and long access sessions.)

Dear Reviewers,

We are grateful to the reviewers for their additional comments. We have provided our responses below in **green** and additional manuscript text is designated in **orange** with manuscript line numbers provided.

REVIEWER COMMENTS

Reviewer #1 (Remarks to the Author):

I appreciate the conscientious response to my earlier critique, but that response has confirmed my reservations about this study and its relevance.
1. Earlier critique and response included the following: "This is by no means comparable to the resolution possible with GWAS mapping in human populations (or presumably even with outbred rats)..." Response: We agree with the reviewer that humans are better for fine mapping. However, there is a trade off ... the greater linkage disequilibrium (LD) observed in HS rats reduces the effective number of hypotheses tested, greatly increasing power, at the expense of mapping precision. This is a key reason that we are able to obtain genome-wide significant results with a sample size that would be underpowered in humans. Because of this decreased resolution, this study is not comparable to a GWAS in humans. As such the title "Genome-wide association study of cocaine self-administration 1 behavior..." does not carry the expected meaning to an investigator familiar with this kind of analyses in humans. A more accurate title would be, "Low-resolution genomewide association study..."
We appreciate the concern and agree that mapping resolution in HS rats differs from humans. People with expertise in the field are aware of these differences. Our title explicitly states that our GWAS is conducted in HS rats. HS rats are notable because they have superior mapping resolution compared to any other available rat population. We have also highlighted these issues in the manuscript. Even among human populations, LD patterns differ such the European-like individuals have lower mapping resolution compared to African-like individuals, which is well known among human geneticists but is essentially never mentioned in the titles of manuscripts.

As the authors acknowledge, this is one of the costs of accomplishing the work and obtaining significant results in a small sample. The loss of resolution is a critical problem and, as the authors confirm elsewhere, compromises identification of specific genes as opposed to relatively broad regions. Ces1 is identified "as a prioritized candidate" and mentioned in the abstract but it is mapped to a region that contains other loci and is not supported with sufficient evidence to be highlighted so strongly.

Similarly the reference to "perfect linkage disequilibrium" is not meaningful because the high levels of LD overall most likely make this the most usual circumstance. I asked the authors to address this question in the previous critique but they did not (see item 4 below). Any reference to "perfect linkage disequilibrium" should be tempered by a consideration of the overall LD in this model.

We agree that the broader LD structure of HS rats limits fine-mapping resolution and previously revised the manuscript to discuss this in the Limitations section. We are careful to describe genes such as *Ces1* as prioritized candidate within associated intervals of high LD. In this case, the associated interval is relatively small (~0.25 mb) and contains a very limited number of genes, including the *Ces1* gene family and one additional gene (Figure 2C). To further support our evidence-based functional prioritization of *Ces1,* we identify multiple coding variants in *Ces1* genes. Given the constrained size of the region and small number of genes in the region, as well as the presence of coding variants, we highlight *Ces1* as a top candidate. Note that even in much larger human populations, statistical fine mapping does not always uniquely identify a single gene.

2. The authors refer to the rodent behavior as "CUD-like". This is not sufficiently supported. It would be more accurate to refer to it as a cocaine consumption behavior – or in some other way that does not draw an explicit parallel to a human diagnosis that the data do not support fully.

The use of the term "CUD-like", or similar phrases, is common in rodent behavior and addiction literature to denote modeling of behavioral features relevant to substance use disorders. We do not claim that HS rats meet criteria for CUD, but rather that the paradigm captures key components of addiction-related behavior. The behaviors measured go well beyond consumption; we show multiple features beyond just consumption such as punished responding and heightened demand as measured using progressive ratio.

3. The lack of a replication for this finding in a small sample remains a serious limitation.

Generating the current cohort of HS rats required nearly a decade of work. We prefer not to delay publication by another decade to generate a replication cohort. The results presented are statistically robust and presented with appropriate caution. Modern human GWAS frequently do not include replication datasets.

4. Item 9 from the first critique "Authors refer to closely-mapped markers in perfect LD (bottom of p10) but considering the LD structure, aren't all closely-mapped markers necessarily in perfect LD? This is relevant to interpretation of the findings and needs to be addressed rigorously" is not responded seriously. This requires a rigorous response.

LD is not uniform across all SNPs. Outbred HS rats were created by crossing eight inbred founders. Thus, there are eight haplotypes, which results in varying levels of LD between pairs of SNPs. Two physically adjacent SNPs are not necessarily in strong LD. In the

context of HS rats, “perfect LD” refers specifically to SNP pairs with $r^2 = 1$ because they represent differences that are characteristic of two non-recombined haplotypes. Outside of the regions with perfect LD, SNPs are not as highly correlated due to recombinations that have occurred in prior generations. This is somewhat analogous to human population isolates like the Amish or Hutterites.

Below, we have provided the regional association plot from Figure 2C, which shows a small region in which some SNPs show high LD with the index SNP (19:13896276) while other show very low levels of LD with the index SNP. In the second column, we provide a zoomed out version of the same region to show that nearby regions have much lower LD with the index SNP. This figure clearly shows that physical proximity and LD are different in HS rats.

For all regions that reached genome-wide significance, our Supplemental Report provides both zoomed-in and zoomed-out regional association plots.

**Figure 2C zoomed-in**	**Figure 2C zoomed-out**
• Zoomed-in region (<1 mb) highlighting the block of SNPs with high LD<br>• Small LD block (~0.25 mb) that encompasses the *Ces1* gene family and one other gene<br>• Red dots represent SNPs that are in high LD ($r^2 > 0.8$)with the top SNP	• Zoomed-out region (6 mb) highlighting adjacent regions that are not in LD with the top SNP<br>• Outside of the small red LD block, there are SNPs that have very low of no correlation with the top SNP (dark blue dots, $r^2 < 0.2$)

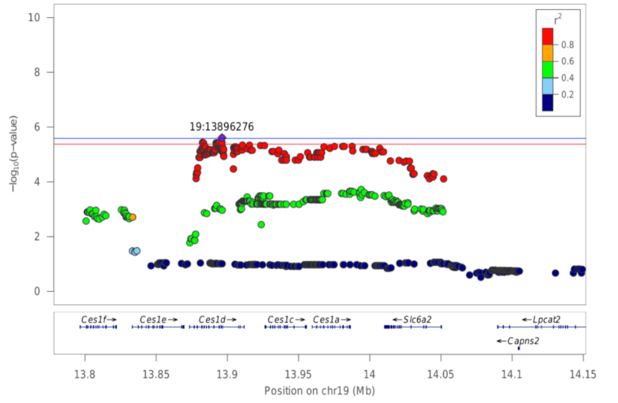

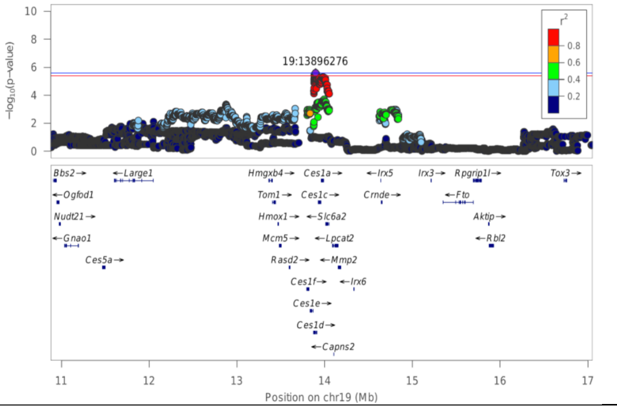

In an effort to address this review’s concerns, our revised manuscript now emphasizes that both versions of these plots are available for all 6 significant associations.

Manuscript Results edit (lines 178 - 179):

> Regional association plots for all significant associations can be found in the Supplementary Report.

Reviewer #2 (Remarks to the Author):

The responses provided and edits performed were generally clear and responsive to the questions raised in the initial review. There is one point to raise at this stage:

In response to the initial review, the authors added text on lines 73-75 that "exposure ... to addictive drugs" would be "unethical in humans". As written, this is incorrect. There are a number of clinical or research reasons why naive people may be exposed to a potentially addictive drug in an ethical manner. This sentence should be substantially revised.

We agree with this comment. Our intent is to highlight the experimental control afforded by animal models. We have edited the intro as follows:

Manuscript Introduction edit (lines 73 - 75):

> In addition, rodent models allow controlled exposure of naïve animals to addictive drugs under tightly-regulated experimental conditions that still capture the extremes of drug-taking behavior.

Reviewer #3 (Remarks to the Author):

I co-reviewed this manuscript with one of the reviewers who provided the listed reports. This is part of the Nature Communications initiative to facilitate training in peer review and to provide appropriate recognition for Early Career Researchers who co-review manuscripts.

We appreciate the consideration and time of these Reviewers.

Reviewer #4 (Remarks to the Author):

The authors have fully answered my questions.

We appreciate the consideration and time of these Reviewers.
